# Evaluation of the Cornea by Anterior Segment Optical Coherence Tomography in Diseases of the Cornea

**DOI:** 10.7759/cureus.70036

**Published:** 2024-09-23

**Authors:** Jhimli Ta, Varsha Manade, Megha R Kotecha, Ozukhil Radhakrishnan, Surbhi A Chodvadiya, Jessica Sangwan

**Affiliations:** 1 Department of Ophthalmology, Dr. D. Y. Patil Medical College, Hospital and Research Centre, Pune, IND

**Keywords:** anterior segment optical coherence tomography (as-oct), corneal diseases, kayser-fleischer (kf) ring, keratoconus suspect, ocular surface squamous neoplasia (ossn)

## Abstract

Aim: The aim of the study was to determine any new findings provided by anterior segment optical coherence tomography (AS-OCT) in different corneal diseases diagnosed by slit lamp examination (SLE).

Methods: This cross-sectional, observational, hospital-based study was conducted at a tertiary care centre in Western Maharashtra from September 2022 to June 2024, and it included 93 eyes of 93 patients with isolated corneal diseases. A detailed SLE of the anterior segment was done to assess corneal pathology, corneal thickness, corneal structural integrity, presence of corneal opacities, corneal vascularization, presence of other abnormalities like corneal degeneration, corneal foreign bodies, Kayser-Fleischer (KF) ring, ocular surface squamous neoplasia (OSSN). All patients underwent AS-OCT with imaging protocols tailored to each corneal condition, including assessment of focal thinning in Keratoconus patients, graft-host junction integrity in post-keratoplasty cases, Descemet’s membrane integrity in post-cataract surgery patients, localization and depth of invasion of corneal foreign bodies, detection of KF rings in Wilson’s disease cases, and identifying and assessing extent of corneal dystrophies, corneal degenerations, and OSSN. AS-OCT findings were compared with SLE results to identify any additional information provided by AS-OCT over SLE in the study population. For statistical analysis, data was entered into Microsoft Excel (Microsoft Corporation, Redmond, Washington, United States) and analysed using IBM SPSS Statistics for Windows, Version 26.0 (Released 2019; IBM Corp; Armonk, New York, United States). Inter-rater agreements between SLE and AS-OCT were evaluated by Cohen’s Kappa by using IBM SPSS Statistics for Windows, Version 26.0.

Results: AS-OCT enabled additional diagnosis of three (15.8%) more cases of keratoconus, which were declared normal on SLE. AS-OCT identified one (5.90%) case of deep-seated stromal corneal foreign body among the cases which were reported to have only superficial corneal foreign body on SLE. Two (50%) cases of suspected OSSN on SLE were ruled out on AS-OCT, whereas two (50%) cases of suspected OSSN on SLE were confirmed on AS-OCT. AS-OCT found a KF ring among two (12.5%) cases that were reported to have no KF ring on SLE. On both SLE and AS-OCT, 26 (81.2%) cases showed the same findings, whereas in 30 (49.2%) cases, AS-OCT rejected the suggested findings of the SLE. In six (18.8%) cases, SLE did not show any findings, but AS-OCT showed findings. Both SLE and AS-OCT showed no findings in 31 (50.8%) cases.

Conclusion: This study highlights the significant role of AS-OCT in enhancing the diagnostic accuracy of various corneal diseases. Its ability to detect additional findings in various corneal conditions such as keratoconus, corneal foreign bodies, OSSN, and KF ring in Wilson’s disease underscores its value in clinical practice. The utility of AS-OCT in diagnosing subclinical corneal diseases which are beyond the scope of routine SLE has been demonstrated in our study. These capabilities make AS-OCT an effective additional diagnostic tool in evaluating cornea and should be routinely incorporated along with traditional SLE in evaluating various corneal diseases.

## Introduction

The evaluation of the cornea is a fundamental aspect of ophthalmology, as the cornea's health and integrity are crucial for maintaining clear vision. Traditionally, slit lamp biomicroscopy has been the cornerstone of corneal examination, offering a detailed view of the corneal surface and anterior chamber. However, the advent of anterior segment optical coherence tomography (AS-OCT) has revolutionized our approach to diagnosing and managing various corneal diseases. AS-OCT is a non-contact and safe procedure that provides high-resolution, cross-sectional imaging of the cornea, offering insights into corneal pathology that are often beyond the reach of conventional slit lamp examination (SLE), as evidenced by a study by Sridhar et al. [1].

Doors et al., in their study, showed that the inception of optical coherence tomography (OCT) dates back to 1991 when it was initially introduced for imaging the posterior segment of the eye [2]. Three years later, the first AS-OCT was proposed as a non-contact and noninvasive imaging technique that captures high-resolution cross-sectional images of the anterior eye segment, as reported in the study by Izatt et al. [3]. A study by Ramos et al. shows that AS-OCT is useful in exploring the anterior segment of the eye [4]. OCT imaging works by measuring the delay of light, typically infrared, as it reflects off tissue structures. It is a valuable non-contact imaging technology utilizing a 1310 nm super luminescent diode. This technology uses a Michelson interferometer, which generates a reference beam of infrared light. It measures multiple other beams of light as they return from the various reflective tissue layers of the eye against this reference beam. There are two main types of AS-OCT: time-domain AS-OCT and Fourier-domain (or spectral-domain) AS-OCT.

## Materials and methods

This was a cross-sectional, observational, hospital-based study with 93 eyes of 93 patients, conducted between September 28, 2022, and June 30, 2024. The study received approval from the Institutional Ethics Committee of Dr. D. Y. Patil Medical College, Hospital and Research Centre, Pune, Maharastra, India (approval number: IESC/PGS/2022/108 dated September 28, 2022). Ethical approval and informed consent procedures were stringently followed, ensuring institutional and international ethical standards adherence. The study was thoroughly discussed with the patients prior to screening and evaluation.

Inclusion and exclusion criteria

All consecutive cases visiting the Ophthalmology Outpatient department at Dr. D. Y. Patil Medical College, Hospital and Research Centre who were diagnosed with isolated corneal diseases on SLE were included in the study. The study excluded patients with isolated conjunctival disorders not involving cornea or significant ocular surface diseases impacting corneal clarity. Patients who were known cases of glaucoma, or had an inability to undergo AS-OCT due to anatomical or technical reasons were excluded from the study. Noncompliant patients and patients not willing to give consent for the study were not included in the study.

Procedure

A detailed ocular and systemic history was taken for all patients, noting demographic factors such as age, gender, family history, and occupation. All patients underwent a comprehensive clinical examination, beginning with best-corrected visual acuity (BCVA) measured on the logMAR scale, followed by an assessment of orbit, adnexa, and extraocular movements.

A detailed SLE of the anterior segment was conducted to evaluate corneal pathology, thickness, structural integrity, opacities, vascularization, and abnormalities such as degeneration, foreign bodies, Kayser Fleischer (KF) ring, and ocular surface squamous neoplasia (OSSN). The detailed findings from the SLE were meticulously recorded for each patient, focusing on identifying the presence, extent, and descriptive findings of the corneal pathologies in all the patients.

Subsequently, AS-OCT was performed in all patients with isolated corneal diseases using CIRRUS™ HD-OCT 500 (Carl Zeiss AG, Oberkochen, Baden-Württemberg, Germany). Proper alignment was ensured and focus was given to obtain high-quality imaging of the corneal structures in various corneal diseases. Cross-sectional images of the cornea were taken to assess corneal thickness, corneal curvature, corneal structural integrity, corneal opacities, presence of pathologies like corneal degeneration, corneal foreign bodies, and KF ring.

AS-OCT imaging protocols were tailored to each corneal condition, including assessment of focal thinning in keratoconus patients, graft thickness, and graft-host junction integrity in post-keratoplasty cases, Descemet’s membrane integrity in post-cataract surgery patients with corneal edema, localization and depth of invasion of corneal foreign bodies, detection of KF rings in Wilson’s disease cases, and identifying and assessing the extent of corneal dystrophies, corneal degenerations, and OSSN. AS-OCT findings were compared with SLE results to identify any additional information provided by AS-OCT over SLE in the study population.

Statistical analysis

For statistical analysis, data was entered in Microsoft Excel (Microsoft Corporation, Redmond, Washington, United States) and analyzed using IBM SPSS Statistics for Windows, Version 26.0 (Released 2019; IBM Corp; Armonk, New York, United States). Qualitative data was expressed in frequency and percentage. Quantitative data was summarized using mean (SD) and median (interquartile range (IQR)) and range (IQR and maximum-minimum values). Age and vision were expressed in mean (SD), median (IQR), and range. Appropriate bar diagrams were made for the results. Inter-rater agreement between SLE and AS-OCT were evaluated by Cohen’s Kappa by using IBM SPSS Statistics for Windows, Version 26.0.

## Results

In the study, out of 93 eyes with isolated corneal diseases who underwent ocular evaluation in the ophthalmology OPD, the corneal diseases observed were as follows: keratoconus in 19 (20.4%) cases, corneal foreign body in 17 (18.3%) cases, suspected graft rejection in keratoplasty postoperative patients in 15 (16.1%) cases, suspected Descemet’s membrane detachment in post-cataract surgery patients in 14 (15.1%) cases, suspected OSSN in four (4.3%) cases, corneal dystrophy in six (6.5%) cases, corneal degeneration in two (2.2%) cases, and suspected KF ring in Wilson’s disease in 16 (17.2%) cases.

Age distribution of the study population was 12-82 years (Table 1). Keratoconus (mean 20.21, range 13-29 years), corneal foreign body (mean 34.18, range 23-48 years), and suspected KF ring in Wilson’s disease showed prevalence in the younger population (mean 21.25, range 12-33 years). Corneal diseases showing prevalence in the older population were suspected Descemet’s membrane detachment in post-cataract surgery patients (mean 65.93, range 53-73 years) and corneal degenerations (58 years). Suspected graft rejection in keratoplasty postoperative patients (mean 54.27, range 13-67 years), Suspected OSSN patients (mean 51.25, range 22-82 years), corneal dystrophy patients (mean 46.67, range 33-62 years) showed prevalence in both young and older population.

**Table 1:** Age distribution of the study population OSSN: ocular surface squamous neoplasia; KF: Kayser Fleischer

Corneal diseases	Mean (SD), years	Median (IQR), years	Minimum-Maximum, years
Keratoconus	20.21 (4.95)	19.00 (17-20)	13-29
Corneal foreign body	34.18 (7.49)	34.00 (29-39.5)	23-48
Suspected postoperative graft rejection in Keratoplasty	54.27 (15.20)	58.00 (52-67)	13-67
Suspected postoperative Descemet’s membrane detachment in cataract surgery	65.93 (4.89)	67.00 (63.5-69.25)	53-73
Suspected OSSN	51.25 (32.69)	50.50 (22.5-80.75)	22-82
Corneal dystrophy	46.67 (12.48)	43.00 (36-62)	33-62
Corneal degeneration	58.00 (0.00)	58.00 (58-58)	58-58
Suspected KF ring in Wilson’s disease	21.25 (7.39)	19.00 (16.75-29.5)	12-33

**Figure 1:**
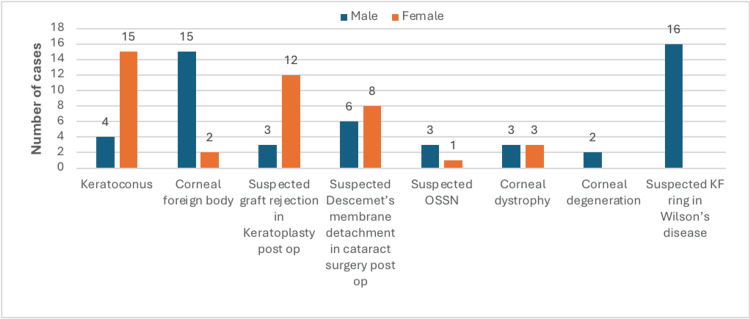
Gender distribution of patients OSSN: ocular surface squamous neoplasia; KF: Kayser Fleischer

Females were the majority among the keratoconus patients with 15 (78.94%) cases, suspected graft rejection in keratoplasty postoperative patients with 12 (80%) cases, suspected Descemet’s membrane detachment in cataract postoperative patients with eight (57.14%) cases. Males were the majority among the corneal foreign body patients with 15 (88.23%) cases, suspected OSSN with three (75%) cases, corneal degeneration with two (100%) cases, and suspected KF ring in Wilson’s disease with 16 (100%) cases. Three (50%) males and three (50%) females were equally distributed among the corneal dystrophy patients (Figure 1).

The range of vision at presentation was documented in LogMAR scale in keratoconus patients (0-0.6), corneal foreign body patients (0-2), suspected graft rejection in post-keratoplasty patients (0-1.7), suspected Descemet’s membrane detachment in post-cataract surgery patients (0.78-1.7), suspected OSSN patients (0-1), corneal dystrophy patients (0.3-0.78), corneal degeneration patients (0.6), and patients with suspected KF ring in Wilson’s Disease (0-0.18) (Table 2).

**Table 2:** Vision at presentation in the study population in LogMAR OSSN: ocular surface squamous neoplasia; KF: Kayser Fleischer

Corneal diseases	Mean (SD)	Median (IQR)	Minimum-Maximum
Keratoconus	0.38 (0.20)	0.30 (0.18-0.60)	0-0.60
Corneal foreign body	0.67 (0.57)	0.48 (0.30-0.78)	0-2
Suspected postoperative graft rejection in keratoplasty	1.29 (0.49)	1.40 (1-1.7)	0-1.7
Suspected postoperative Descemet’s membrane detachment in cataract surgery	1.14 (0.31)	1.00 (0.95-1.4)	0.78-1.7
Suspected OSSN	0.64 (0.44)	0.78 (0.2-0.95)	0-1
Corneal dystrophy	0.64 (0.19)	0.69 (0.53-0.78)	0.3-0.78
Corneal degeneration	0.60 (0.00)	0.60 (0.60)	0.60
Suspected KF ring in Wilson’s disease	0.03 (0.07)	0.00 (0)	0-0.18

In the study, the provisional diagnosis given by SLE were as follows: keratoconus in 16 (17.2%) cases, suspected keratoconus in three (3.2%) cases, superficial corneal foreign body in 17 (18.3%) cases, suspected graft rejection in post-keratoplasty surgery patients in 15 (16.1%) cases, suspected Descemet’s membrane detachment in post-cataract surgery patients in 14 (15.1%) cases, suspected OSSN in four (4.3%) cases, corneal dystrophy (granular dystrophy in four (4.3%) cases and Fuch’s dystrophy in two (2.2%) cases), Terrien’s marginal degeneration in two (2.2%) cases, Wilson’s disease with no KF Ring in 16 (17.2%) cases (Table 3).

**Table 3:** Distribution of provisional diagnosis of the study population on slit lamp examination (N=93) OSSN: ocular surface squamous neoplasia; KF: Kayser Fleischer

Corneal diseases	Frequency (Percentage)
Keratoconus	16 (17.2%)
Suspected Keratoconus	3 (3.2%)
Superficial corneal foreign body	17 (18.3%)
Suspected postoperative graft rejection in keratoplasty	15 (16.1%)
Suspected postoperative Descemet’s membrane detachment in cataract surgery	14 (15.1%)
Suspected OSSN	4 (4.3%)
Corneal dystrophy (Granular dystrophy)	4 (4.3%)
Corneal dystrophy (Fuch's dystrophy)	2 (2.2%)
Terrien’s marginal degeneration	2 (2.2%)
Wilson's disease with no KF ring	16 (17.2%)

The final diagnosis given by AS-OCT was keratoconus in 19 (20.4%) cases, superficial corneal foreign body in 16 (17.2%) cases, superficial with deep-seated corneal foreign body in one (1.1%) case, no graft rejection in keratoplasty postoperative patients in 15 (16.1%) cases, no Descemet’s membrane detachment in post-cataract surgery patients in 14 (15.1%) cases, no OSSN in two (2.2%) cases, OSSN in two (2.2%) cases, corneal dystrophy (granular dystrophy in four (4.3%) cases and Fuch’s dystrophy in two (2.2%) cases), Terrien’s marginal degeneration in two (2.2%) cases, Wilson’s disease with no KF ring in 14 (15.1%) cases, and Wilson’s disease with KF ring in two (2.2%) cases (Table 4).

**Table 4:** Distribution of final diagnosis of the study population on AS-OCT (N=93) OSSN: ocular surface squamous neoplasia; KF: Kayser Fleischer; AS-OCT: anterior segment optical coherence tomography

Corneal diseases	Frequency (Percentage)
Keratoconus	19 (20.4%)
Superficial corneal foreign body	16 (17.2%)
Superficial epithelial corneal foreign body with deep-seated corneal stromal foreign body	1 (1.1%)
No postoperative graft rejection in keratoplasty	15 (16.1%)
No postoperative Descemet’s membrane detachment in cataract surgery	14 (15.1%)
No OSSN	2 (2.2%)
OSSN	2 (2.2%)
Corneal dystrophy (granular dystrophy)	4 (4.3%)
Corneal dystrophy (Fuch's dystrophy)	2 (2.2%)
Terrien’s marginal degeneration	2 (2.2%)
Wilson's Disease with no KF Ring	14 (15.1%)
Wilson's disease with KF ring	2 (2.2%)

AS-OCT enabled the additional diagnosis of three (15.8%) more cases of keratoconus, which were declared normal on SLE. In all other 16 (84.2%) cases of keratoconus, SLE findings suggestive of Keratoconus were confirmed on AS-OCT (Table 5).

**Table 5:** Additional findings of AS-OCT over slit lamp examination in suspected Keratoconus patients AS-OCT: anterior segment optical coherence tomography

	Keratoconus +	Keratoconus -
Slit lamp examination	16 (84.2%)	3 (15.8%)
AS-OCT	19 (100%)	0 (0%)

AS-OCT identified one (5.90%) case of deep-seated corneal stromal foreign body along with superficial corneal foreign body among the 17 (100%) cases that were reported to have only superficial corneal foreign body on SLE (Table 6).

**Table 6:** Additional findings of AS-OCT over slit lamp examination in eyes with corneal foreign body AS-OCT: anterior segment optical coherence tomography

	Superficial epithelial corneal foreign body	Superficial epithelial corneal foreign body with deep-seated corneal stromal foreign body
Slit lamp examination	17 (100%)	0 (0%)
AS-OCT	16 (94.10%)	1 (5.90%)

In two (50%) cases of suspected OSSN on SLE, AS-OCT found no corneal epithelium thickening, hyper-reflectivity, or abrupt transition indicating no OSSN. Two (50%) other suspected cases of OSSN on SLE were found to have corneal epithelium thickening with hyper-reflectivity and abrupt transition (OSSN) on AS-OCT thus confirming the diagnosis of OSSN (Table 7).

**Table 7:** Additional findings of AS-OCT over slit lamp examination in suspected OSSN patients AS-OCT: anterior segment optical coherence tomography; OSSN: ocular surface squamous neoplasia

	OSSN	No OSSN
Slit lamp examination	4 (100%)	0 (0%)
AS-OCT	2 (50%)	2 (50%)

AS-OCT found KF rings in two (12.5%) out of the 16 (100%) cases that were reported to have no KF ring on SLE (Table 8).

**Table 8:** Additional findings of AS-OCT over slit lamp examination in suspected KF ring in Wilson’s disease patients. AS-OCT: anterior segment optical coherence tomography; KF: Kayser-Fleischer

	Normal (No KF ring seen)	KF ring
Slit lamp examination	16 (100%)	0 (0%)
AS-OCT	14 (87.5%)	2 (12.5%)

AS-OCT ruled out graft rejection in all suspected graft rejection post-keratoplasty patients (n=15; 100%) who were considered suspects after SLE. AS-OCT also ruled out Descemet’s membrane detachment in all suspected Descemet’s membrane detachment post-cataract surgery patients (n=14; 100%) who were considered suspects on SLE. 

SLE diagnosed patients with corneal dystrophies as granular dystrophy in four (4.3%) cases and Fuch’s dystrophy in two (2.2%) cases, the findings of which were confirmed on AS-OCT. Two (2.2%) cases of corneal degeneration were diagnosed as Terrien’s marginal degeneration on SLE, the findings of which were confirmed on AS-OCT.

In both SLE and AS-OCT, 26 (81.2%) cases showed the same findings, whereas in 30 (49.2%) cases, AS-OCT rejected the findings suggested by SLE (Table 9). In six (18.8%) cases, SLE did not show any findings but AS-OCT showed findings. Both SLE and AS-OCT showed no findings in 31 (50.8%) cases. Agreement in the diagnosis between the SLE and AS-OCT was determined by Cohen’s Kappa. The value of Kappa in the study was 0.272 (p=0.003). A significant, but minimal agreement, was found between SLE and AS-OCT.

**Table 9:** Slit lamp examination vs AS-OCT findings in the study population AS-OCT: anterior segment optical coherence tomography

Slit lamp examination	AS-OCT		Total, n
	Yes, n (%)	No, n (%)	
Yes	26 (81.2%)	30 (49.2%)	56
No	6 (18.8%)	31 (50.8%)	37
Total	32	61	93

## Discussion

OCT, originally designed for imaging the posterior aspect of the eye, has proven to be highly effective in thoroughly capturing images of the ocular surface and the anterior segment in a front-to-back sequence. AS-OCT is becoming an important tool in the evaluation of cornea. Sridhar et al., in their study, demonstrated that AS-OCT can be of paramount importance in various corneal pathologies such as Descemet’s membrane assessment, corneal dystrophies, evaluation of corneal graft after keratoplasty surgery, diagnosis of early-stage surface neoplasia [1]. AS-OCT is a valuable non-contact imaging technology which aids the clinician to see corneal pathology better when the slit-lamp biomicroscope is unable to give enough details.

**Figure 2:**
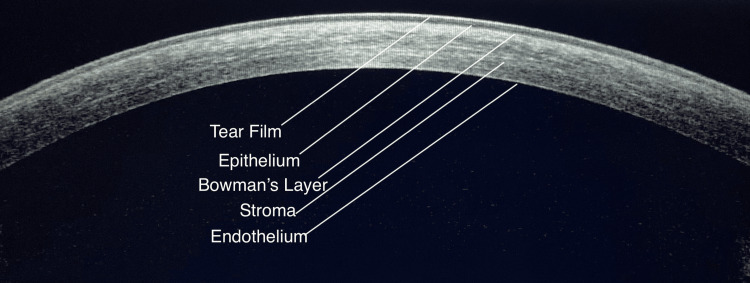
Anterior segment optical coherence tomography of normal cornea.

A horizontal OCT scan of a healthy cornea reveals a highly reflective tear film overlying the epithelium, Bowman's layer, stromal layer, Descemet's membrane, and endothelium (Figure 2). AS-OCT can evaluate a wide range of anterior segment parameters and has several applications in various ocular conditions.

The study was undertaken in a clinical setting of a tertiary care facility in western India to explore the utility of the AS-OCT over the SLE in determining the diagnosis of various corneal diseases and conditions, identify additional findings provided by AS-OCT that are not detectable in SLE. The corneal diseases observed were keratoconus in 19 (20.4%) cases, corneal foreign body in 17 (18.3%) cases, suspected graft rejection in keratoplasty post-operative patients in 15 (16.1%) cases, suspected Descemet’s membrane detachment in post-cataract surgery patients in 14 (15.1%) cases, suspected OSSN in four (4.3%) cases, corneal dystrophy in six (6.5%) cases, corneal degeneration in two (2.2%) cases, and suspected KF ring in Wilson’s disease in 16 (17.2%) cases.

In the current study, distinct gender patterns were seen among various corneal diseases. Females predominated in cases of Keratoconus with 15 (78.9%) cases, suspected graft rejection in keratoplasty postoperative patients with 12 (80%) cases, and suspected Descemet’s membrane detachment in post-cataract surgery patients with eight (57.1%) cases. Conversely, males were more frequently affected by corneal foreign bodies (n=15; 88.2%), OSSN (n=3; 75%), and KF ring in Wilson's disease (n=16; 100%). Corneal dystrophy exhibited equal distribution between genders.

In this study, 19 patients of keratoconus were included, among which females were the majority with 15 (78.9%) cases. Kim et al., in their study of keratoconus patients, also had a majority of female patients (53.5%) [5]. Males were the majority among the corneal foreign body patients (88.2%) in the current study. Akbas et al. [6] and Wang et al. [7] also reported similar gender prevalence in their studies (80% and 75% males, respectively). This is in line with the general epidemiology of the morbidity. In a study by Yenerel et al., males (72.4%) were the majority among the post keratoplasty patients studied [8]. In the current study, males were the majority among the OSSN patients with three (75%) cases. Karp et al., in their study among eight patients with OSSN who underwent surgical removal, assessed the utility of AS-OCT in demarcating the tumor margins [9]. They also had a majority of male patients (87.5%).

Three (50%) males and three (50%) females were equally distributed among the corneal dystrophy patients reported in this study. Granular dystrophy, followed by Fuch’s dystrophy was the major diagnosis in this study. Siebelmann et al., in their study exploring the features of corneal dystrophies on AS-OCT, also reported a similar sex predilection [10]. Two (100%) cases of corneal degeneration were included in this study, both were males. In this study, 16 (100%) patients with Wilson’s disease were included, all of whom were males.

In a study from Hyderabad, India, Sridhar et al. compared the utility of AS-OCT with Slit lamp examination, among seven patients of Wilson’s disease with KF rings [11]. Ormeci et al. from Turkey, included a relatively larger sample of 64 Wilson’s disease patients to compare the diagnostic capability of SLE and AS-OCT [12]. While males were the majority in the Sridhar et al. [11] and Ormeci et al. [12] studies, they included females as well in their study.

In this study, the age range of the study population varied widely (12-82 years). Keratoconus and KF ring in Wilson's disease predominantly affected younger individuals (13-29 and 12-33 years, respectively). Older age groups presented with conditions such as suspected Descemet’s membrane detachment (53-73 years) and Corneal degeneration (58 years). The mean age of the Keratoconus patients was 20.21 years. Kim et al., in their study, included relatively older patients (range 21-42 years, mean 30.95 years) [5]. In the current study, patients with corneal foreign bodies had a mean age of 34.18 years. Akbas et al. [6] and Wang et al. [7] also included patients of similar age in their study (mean 34.9 years and 37.8 years, respectively).

The mean age of the keratoplasty postoperative patients was 54.27 years in this study, while the mean age of patients included in Yenerel et al.'s study was lower (mean=42.7 years) [8]. The mean age of suspected Descemet’s membrane detachment patients was 65.93 years in the present study. Patients included in the study by Zhou et al. were of a relatively younger age than this study (mean=45.57 years) [13]. The older age group of patients in our study might be because we included postoperative cataract patients (a condition itself is associated with older age), while Zhou et al. included patients with varied etiology [13].

The mean age of the OSSN patients in this study was 51.25 years, which was younger than the patients included in the study by Karp et al. (mean=67 years) [9]. While the mean age of the corneal dystrophy patients was 46.67 years in our study, Siebelmann et al., in their study, showed that adolescence was the period of common occurrence [10]. This difference in the age might be due to the delay in presentation in our study. The mean age of the patients with Wilson’s disease in this study was 21.25 years with a range of 12-33 years. Sridhar et al. also included patients of similar age with a range of 8-40 years in their study [11].

Additional diagnostic capability of AS-OCT

In this study, the additional diagnostic capability of AS-OCT over SLE was evident in several conditions.

Keratoconus

**Figure 3:**
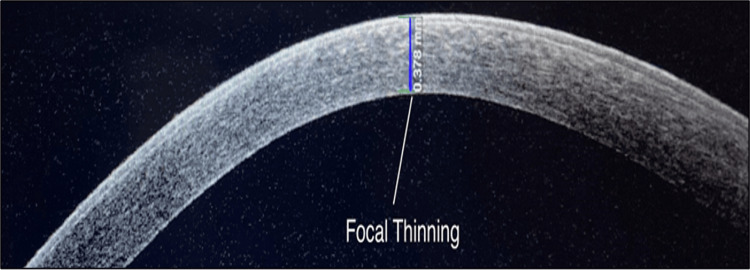
AS-OCT image of keratoconus eye showing focal thinning of cornea AS-OCT: anterior segment optical coherence tomography

Keratoconus, the most common ectatic disorder, is characterized by bilateral and progressive corneal thinning and apical protrusion as evidenced in the study by Sridhar et al. [1]. In general, focal thinning occurs in the inferotemporal corneal location, and detecting this characteristic corneal thinning pattern is a useful new approach to diagnosing keratoconus as evidenced by studies [1,14] (Figure 3).

In our study, AS-OCT identified three (15.8%) additional cases of keratoconus that were missed by SLE, highlighting its sensitivity in detecting early or subtle corneal changes. All three patients showed focal thinning on AS-OCT. In all other cases, SLE findings suggestive of keratoconus were confirmed on AS-OCT. Kim et al. in their study reported AS-OCT as an effective measure to diagnose and demarcate keratoconus from the normal and suspected patients [5].

Corneal Foreign Body

**Figure 4:**
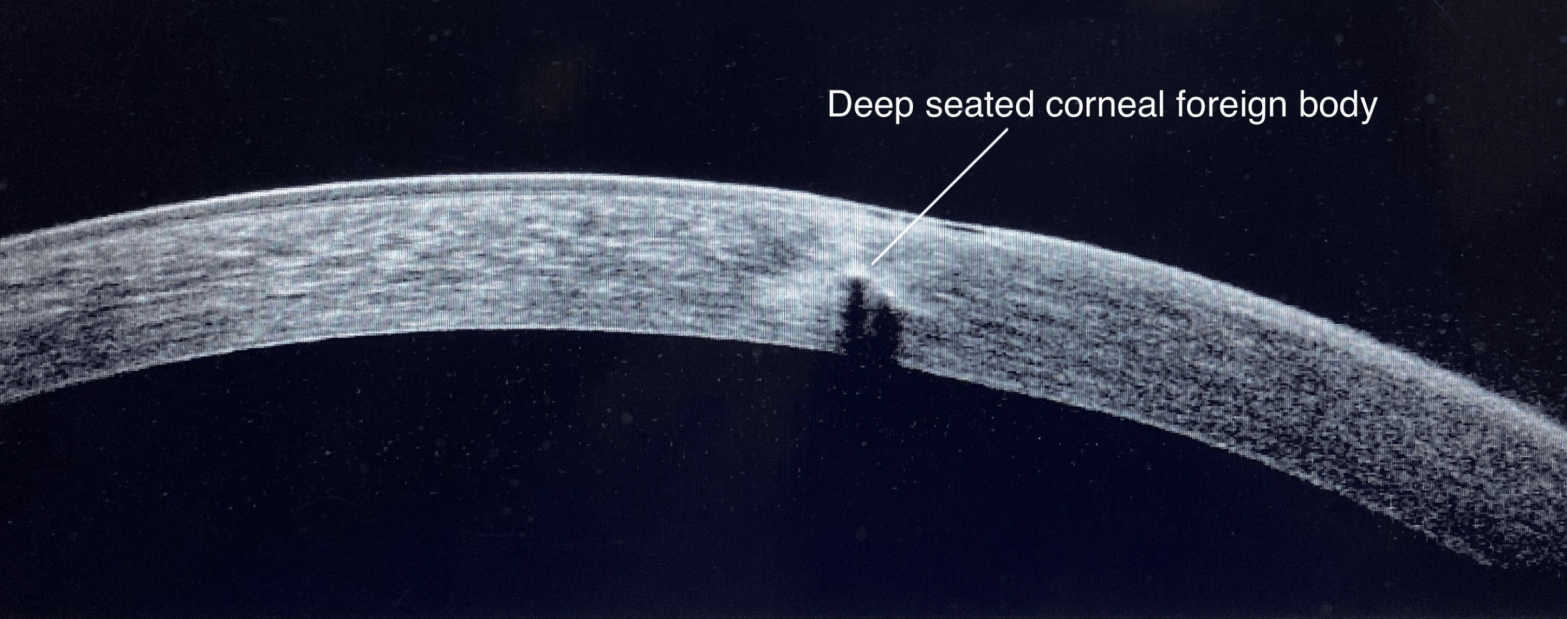
AS-OCT imaging showing a deep-seated corneal foreign body AS-OCT: anterior segment optical coherence tomography

Most emergency visits for eye injuries are attributed to corneal foreign bodies, which are typically associated with pain as evidenced by a study by McGwin et al. [15]. In the current study, 17 patients with corneal foreign bodies were evaluated using both SLE as well as AS-OCT. AS-OCT detected one (5.90%) case of a deep-seated stromal foreign body among the 17 (100%) cases initially identified as only superficial corneal foreign bodies, demonstrating its utility in the precise localization of foreign bodies (Figure 4). On AS-OCT images, Wang et al. in their study found that foreign bodies displayed distinctive features, including a high signal with subsequent shadowing at the central region and a low signal with ensuing shadowing at the margins [7].

In their study, Akbas et al. reported that AS-OCT could identify the majority of the foreign body types except for the chestnut burr, owing to its organic nature [6]. Also, the location of the foreign body was not reported in their study, which could be determined in this study. Corneal foreign bodies which are transparent and are inserted in the deeper regions can be especially challenging to evaluate as evidenced in the study by Arora et al. [16], at which points AS-OCT can be useful, as shown in this study. 

OSSN

**Figure 5:**
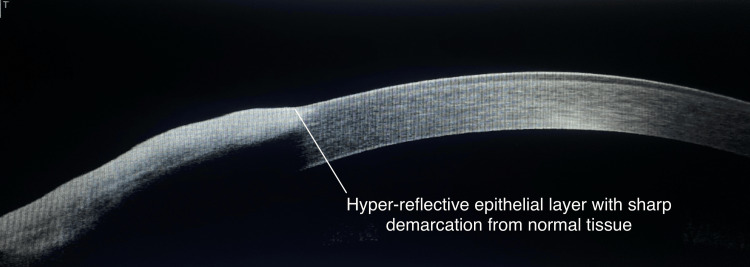
AS-OCT imaging of OSSN showing a thickened, hyper-reflective epithelial layer, sharp demarcation from normal tissue, increased epithelial thickness, disrupted normal architecture, and invasion into the cornea AS-OCT: anterior segment optical coherence tomography; OSSN: ocular surface squamous neoplasia

In this study, AS-OCT provided crucial information about corneal epithelium thickening and hyper-reflectivity, distinguishing between true OSSN cases and other conjunctival lesions involving the cornea, thus aiding in accurate diagnosis and management (Figure 5).

Karp et al., in their study among eight patients with OSSN who underwent surgical removal, assessed the utility of OSSN in demarcating the tumor margins [9]. In this study, two (50%) cases of suspected OSSN, were found to have no corneal epithelium thickening, hyper-reflectivity, or abrupt transition seen on AS-OCT. Two (50%) other cases in our study which were suspected to be OSSN were confirmed on AS-OCT. Using optical methods like AS-OCT to identify tumor edges could potentially lower the rate of remaining positive margins in OSSN and reduce the excision of healthy tissue as shown in the study by Karp et al. [9].

KF Ring in Wilson's Disease

**Figure 6:**
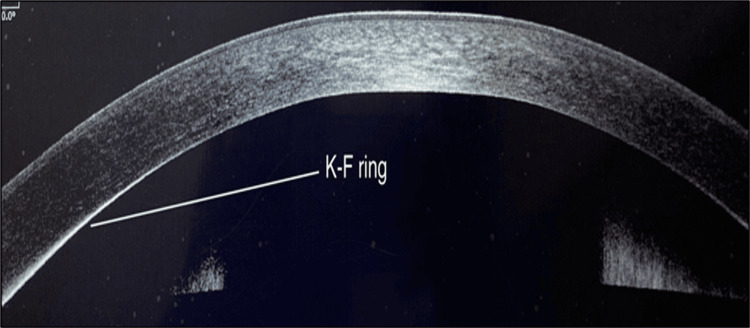
AS-OCT imaging showing hyperreflective line at the level of Descemet’s membrane (KF ring) in a case of Wilson’s disease. AS-OCT: anterior segment optical coherence tomography; KF: Kayser Fleischer

KF ring was seen as hyper-reflectivity at the level of Descemet's membrane in the peripheral cornea as shown in the study by Sridhar et al. [11] (Figure 6). In our study among Wilson’s disease patients, AS-OCT identified hyper-reflective bands at the level of Descemet’s membrane confirming the presence of KF ring in two (12.5%) cases, initially reported to be negative on SLE, proving the effectiveness of AS-OCT in detecting early or subtle KF rings.

In the study by Ormeci et al., 34.4% of the overall Wilson’s disease patients showed KF findings [12]. Of the patients, 15.4% were positive for KF ring in both SLE and AS-OCT assessment, indicating a significantly greater number of KF ring-positive patients identified through AS-OCT.

In a study from Hyderabad, India, Sridhar et al. compared the utility of AS-OCT with SLE, among seven patients of Wilson’s disease with KF rings [11]. They demonstrated that AS-OCT can be used as an alternative method of evaluating KF ring in Wilson’s disease by not just ophthalmologists but by other clinicians handling patients with Wilson’s disease as well.

Post-Keratoplasty Patients

**Figure 7:**
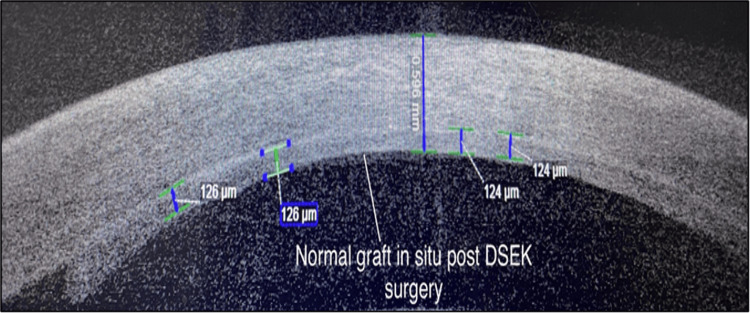
AS-OCT imaging of postoperative keratoplasty (DSEK) eye showing normal graft in situ with no signs of graft rejection or graft dislocation AS-OCT: anterior segment optical coherence tomography; DSEK: Descemet’s stripping endothelial keratoplasty

AS-OCT can be used to assess graft thickness, graft integrity, and graft host junction reaction in post-keratoplasty patients (Figure 7). In this study, 15 keratoplasty postoperative patients who were suspected to have graft rejection in post keratoplasty patients were evaluated. All 15 (100%) patients in this study were found to have healthy graft host junction on AS-OCT, indicating its potential utility in ruling out suspected graft rejections in post-keratoplasty patients. Yenerel et al. from Turkey assessed the role of AS-OCT in assessing the outcomes following keratoplasty among 58 patients [8]. They reported three graft rejections among their patients, while none were found to have graft rejection in our study.

Descemet’s Membrane Detachment

In this study, the AS-OCT examination ruled out the presence of Descemet’s membrane detachment among all cases reported as a suspect on SLE. This underscores the high utility of AS-OCT in ensuring prudent diagnosis of Descemet’s membrane detachment and aids in early management. Zhou et al. in their study assessed the diagnostic as well as therapeutic usefulness of AS-OCT among seven patients with Descemet’s membrane detachment and found that AS-OCT demonstrated Descemet’s membrane detachment among all their patients, with specific patterns present in each of these cases [13]. In addition to this, they also brought out the utility of the AS-OCT in the treatment monitoring of Descemet’s membrane detachment patients. 

Corneal Dystrophy

**Figure 8:**
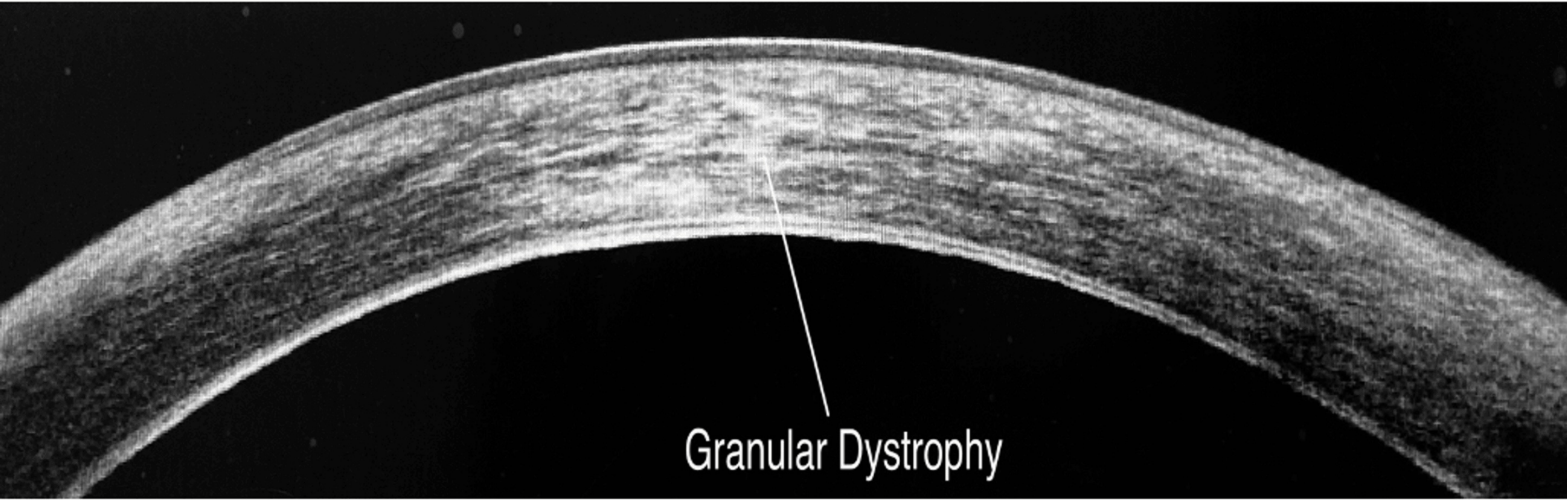
AS-OCT imaging of a patient with corneal dystrophy showing granular dystrophy at the level of stroma AS-OCT: anterior segment optical coherence tomography

In this study, SLE diagnosed corneal dystrophies as granular dystrophy in four (4.3%) cases and as Fuch’s dystrophy in two (2.2%) cases, the findings of which were confirmed on AS-OCT. In patients with Granular Corneal dystrophy, Slit lamp examination revealed multiple white and greyish corneal opacities resembling breadcrumbs or snowflakes. These findings were confirmed on AS-OCT which showed hyperreflective opacities at the anterior stroma of cornea (Figure 8).

SLE in patients with Fuch’s dystrophy showed the presence of multiple corneal guttae which appeared as small excrescences or bumps on retroillumination. AS-OCT revealed hyperreflective nodules on the endothelial surface corresponding to corneal guttae and this confirmed the diagnosis. Thus AS-OCT gave more detailed imaging of the corneal dystrophies revealing hyper-reflective deposits at the anterior stromal epithelium level or the endothelium level. 

Siebelmann et al. in his study showed that AS-OCT enabled detection of the depth, extent, and distribution of pathologies in different corneal layers in patients with corneal dystrophies [10]. This information further helps to make decisions between superficial laser treatment and corneal surgery. Siebelmann et al. also reported that epithelial dystrophies showed non-regular features in the basement membrane [10]. Their study also showed that stromal dystrophies were found to have hyperreflective contents at the Bowman layer, which were also thick.

Corneal Degeneration

**Figure 9:**
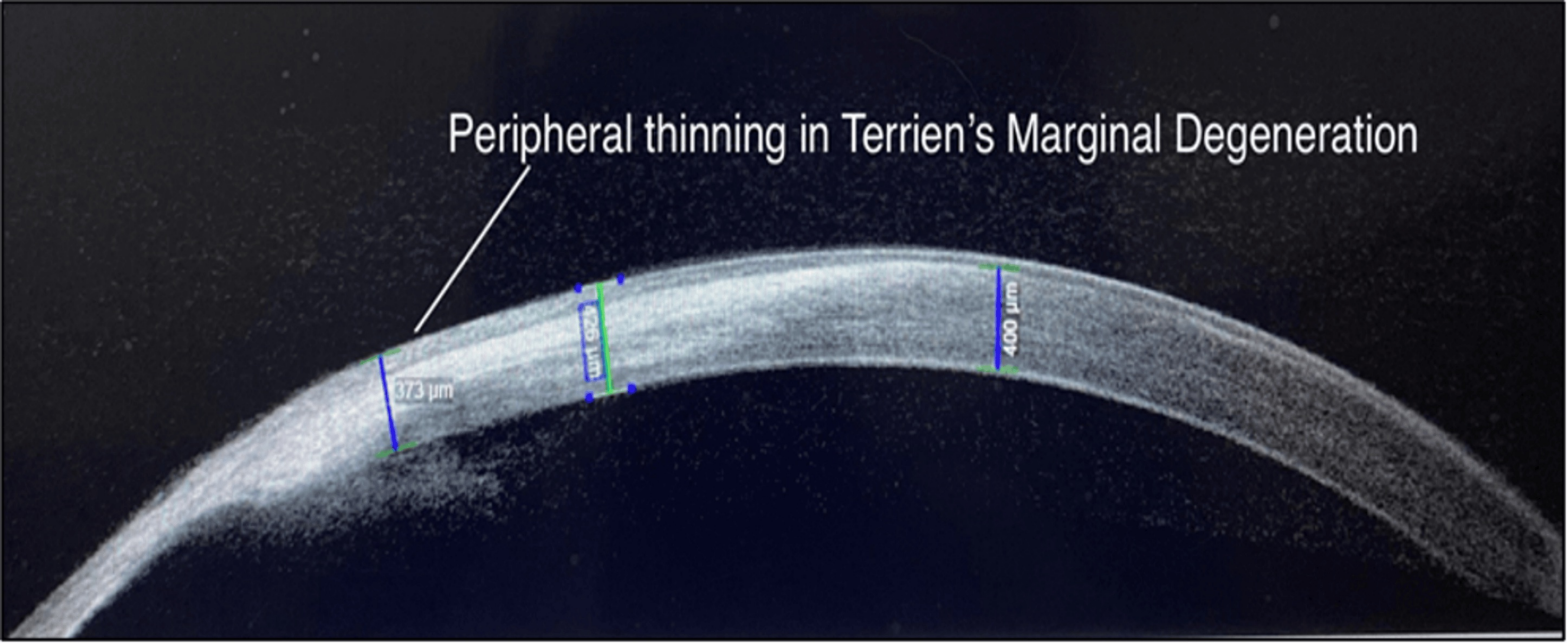
AS-OCT imaging of Terrien’s marginal degeneration AS-OCT: anterior segment optical coherence tomography

Two cases of corneal degeneration were included in our study, in which peripheral thinning and steepening without any signs of inflammation were identified on SLE. A diagnosis of Terrien’s marginal degeneration was made based on SLE findings which was confirmed on AS-OCT. AS-OCT showed high-resolution cross-sectional image of peripheral corneal thinning with a clear delineation of the steepened edge and no signs of inflammation, thus confirming the diagnosis of Terrien’s marginal degeneration (Figure 9). Intrastromal clefts with Descemet’s membrane detachment was seen on AS-OCT in a previous study by Vanathi et al. [17] in the marginal corneal degeneration cases.

These findings demonstrate the ability of AS-OCT to enhance diagnostic precision and improve patient management in various corneal pathologies.

Limitations

The study also had various limitations. The sample size was small since multiple corneal conditions were studied. Owing to the inclusion of patients attending a single institute, the external validity of our findings was limited. The utility of AS-OCT in the therapeutic aspects (such as changes in the condition following treatment) was not assessed in the current study. There was no follow-up of patients in our study.

## Conclusions

The study highlights the significant role of AS-OCT in enhancing the diagnostic accuracy of various corneal diseases. The superior diagnostic accuracy of AS-OCT over SLE has been demonstrated in this study, particularly in identifying conditions like keratoconus, differentiating between superficial and deep-seated corneal foreign bodies, and detecting subclinical cases of KF ring in Wilson's disease and OSSN. AS-OCT also helped us to rule out graft rejection in suspected graft rejection cases post Keratoplasty surgery and Descemet’s membrane detachment in suspected DMD post-cataract surgery patients. This underscores the critical role of AS-OCT in enhancing diagnostic precision and improving patient management in various corneal pathologies.

These capabilities make AS-OCT an effective additional diagnostic tool that complements SLE, enhancing the overall assessment and management of corneal diseases. Further, multi-centric studies with large sample sizes and evaluating the therapeutic aspects of monitoring corneal diseases post treatment must be undertaken to improve the external validity and generalisability of the findings.
